# Age-related decline in cognitive control: the role of fluid intelligence and processing speed

**DOI:** 10.1186/1471-2202-15-7

**Published:** 2014-01-08

**Authors:** Marine Manard, Delphine Carabin, Mathieu Jaspar, Fabienne Collette

**Affiliations:** 1 Cyclotron Research Centre, University of Liège, Liège, Belgium; 2 Department of Psychology: Cognition and Behavior, University of Liège, Boulevard du Rectorat 3, Bâtiment B33, B-4000 Liège, Belgium; 3 Belgian National Fund for Scientific Research (FRS-FNRS), 1000 Brussels, Belgium

**Keywords:** Cognitive control, Aging, Fluid intelligence, Processing speed, Working memory

## Abstract

**Background:**

Research on cognitive control suggests an age-related decline in proactive control abilities whereas reactive control seems to remain intact. However, the reason of the differential age effect on cognitive control efficiency is still unclear. This study investigated the potential influence of fluid intelligence and processing speed on the selective age-related decline in proactive control. Eighty young and 80 healthy older adults were included in this study. The participants were submitted to a working memory recognition paradigm, assessing proactive and reactive cognitive control by manipulating the interference level across items.

**Results:**

Repeated measures ANOVAs and hierarchical linear regressions indicated that the ability to appropriately use cognitive control processes during aging seems to be at least partially affected by the amount of available cognitive resources (assessed by fluid intelligence and processing speed abilities).

**Conclusions:**

This study highlights the potential role of cognitive resources on the selective age-related decline in proactive control, suggesting the importance of a more exhaustive approach considering the confounding variables during cognitive control assessment.

## Background

Cognitive control is a fundamental aspect of cognition. This ability is required to adjust and flexibly guide people’s behavior in changing environmental circumstances, especially in situations where distracting information or a predominant response tendency must be ignored in order to successfully act in a goal-oriented manner. The notion of “cognitive control” can be conceived as a global term that encompasses such well-known psychological concepts as executive/attentional control, goal maintenance, top-down processing, response selection and response inhibition.

Cognitive control was first highlighted by effects such as the adaptation to conflict or the proportion congruence ([1] for discussion of these effects). The adaptation to conflict effect (the “Gratton effect”; [2]) refers to a decrease of the interference effect for items following incongruent ones by comparison to those following congruent ones, as well as a slowing down of the processing of congruent events following incongruent ones. The proportion congruence effect reflects how task context can influence performance. Classically, this effect is investigated through paradigms varying the amount of interference within a task (for example; the Stroop task [3], the Eriksen flanker task [4], the Simon task [1,5,6] or the probe recency task [7]) and refers to the observation of smaller interference effects in lists of stimuli including mainly incongruent items (low-proportion-congruent condition) than in lists including mainly congruent trials (high-proportion-congruent condition [8,9].

Several models of cognitive control have been proposed. Among them, the information processing model [9,10] suggests that interference produced by the simultaneous processing of relevant and irrelevant response pathways might be overcome by a “task demand unit” (corresponding to top-down control) that favors the processing in the appropriate pathway. The conflict monitoring theory [11,12] refined that model by including the influence of task context to explain interference effects. Indeed, the conflict monitoring theory proposes that performance is continuously adjusted during the task according to the amount of conflict previously encountered. Finally, the importance of active maintenance of memory representations (plan of action, goals, or task-relevant information) to overcome predominant but inappropriate responses was stressed by Kane and Engle [13].

In accordance with these models, Braver and colleagues [14] developed a “context-processing” account to provide an explanation of the previously observed age-related decline in cognitive functions [15-19]. These authors defined “context” as all internal task-relevant representations (based on a particular previous stimulus, the processing of an entire sequence of stimuli, specific task instructions, or a particular goal) allowing to bias behavior to efficiently respond to task demands. Consequently, context representations may be particularly important to influence cognitive processing. Braver et al. [14] argued that age-related difficulties observed in working memory, inhibition, attention and executive function may be in fact influenced by the impairment of the ability to maintain context representations in an active state. Following this theoretical background, Braver and collaborators [20] also described a general framework of cognitive control (“the Dual Mechanisms of Control model”, DMC, see below for a detailed presentation) that postulates the existence of two distinct mechanisms: proactive and reactive control processes see also [21,22]. In the present study, we were interested in investigating the potential age-related cognitive control decline by distinguishing proactive and reactive control processes. Indeed, the DMC account provides an elegant framework to subtly assess individual differences in cognitive control processes and complements the “context processing theory” [13,14].

### The Dual Mechanisms of Control (DMC) account

The Dual Mechanism of Control (DMC) account [20] states that flexibility in cognitive control strategies may be achieved through reactive or proactive control, depending on situational demands or individual differences. The DMC model clearly distinguishes these two kinds of control in terms of cognitive properties and brain activity. Proactive control is postulated to be a sustained form of control that can be engaged in situations in which upcoming stimuli can be anticipated, allowing for rapid and efficient responses. More specifically, proactive control involves active maintenance of all task-relevant information (i.e., task instructions, identity of previous stimuli, cues for later behavior, etc.) that could be useful to produce an appropriate response to cognitively demanding events. Reactive control, on the other hand, is thought to be engaged in situations in which anticipating the characteristics of upcoming stimuli is not the most efficient way to perform the task. In that case, the occurrence of a critical event triggers the transient reactivation of required information, specifically in response to that critical stimulus. In sum, proactive control mechanisms are specialized in anticipating and preventing interference, whereas reactive control is dedicated to detecting and resolving interference whenever it occurs [21].

As mentioned earlier, an important factor that may modulate the extent to which proactive or reactive strategies contribute to task performance is the overall task context (i.e., task demands and characteristics). Indeed, although both strategies are equally likely to lead to correct performance on a specific trial, there are some situations in which one or the other kind of control is most appropriate, and the task context encourages the adoption of one form of control over the other. Among these factors, conditions involving high interference levels and allowing the anticipation of interference should encourage the use of proactive control whereas situations in which interference is infrequent and unexpected, reactive control mechanisms are predicted to dominate [20].

Recent data supporting that distinction between proactive and reactive cognitive control have notably been obtained with the Stroop and the probe recency tasks. For example, Bélanger et al. [3] investigated these two processes by manipulating proportions of congruent items within a Stroop task. The comparison of performance between congruent and incongruent trials within the mostly congruent condition was assumed to reflect interference resolution abilities or the involvement of reactive control processes while the comparison of performance between incongruent trials of the mostly congruent and the mostly incongruent conditions was supposed to reflect the contribution of proactive control by the involvement of goal maintenance abilities. As suggested by the DMC model, Bélanger et al. [3] observed that young participants were slower and less accurate for incongruent trials than congruent ones in the mostly congruent condition. Moreover, they were also slower and less accurate for the incongruent trials that occurred during the mostly congruent condition than for the ones presented in the mostly incongruent condition.

The implementation of proactive and reactive control processes was also investigated by Burgess and Braver [7] using the Sternberg probe recency task [23]. In this task, sets of items are presented, followed after a brief delay by a single probe item. Participants have to indicate whether the probe item was part of the memory set (positive probe) or not (negative probe). However, in some trials, the probe was also “recent,” meaning that it had been presented during the memory set in the prior trial, leading to a higher degree of interference in the current trial. As expected, Burgess and Braver ([7], pilot study) observed that the interference associated with negative probes (more errors and slower response times) tended to be lower in the high- than in the low-interference expectancy condition, indicating the recruitment of proactive control processes to efficiently cope with a high-interference context.

According to Braver and colleagues [20,21], another important factor that likely influence the selection of a control strategy is individual differences in cognitive abilities. Because proactive control is more resource-demanding, the implementation of such control when required by task characteristics will be more efficient for individuals who have more cognitive resources available. In fact, impairment in cognitive control previously reported in pathological or developmental populations (e.g., individuals with schizophrenia, older adults, etc.) might stem from differential reliance on reactive and proactive control processes. Consequently, the influence of three factors (age, level of fluid intelligence and processing speed) on the selection of these strategies will be investigated in the present study.

### Fluid intelligence, processing speed and cognitive control

#### Fluid intelligence and cognitive control

As mentioned above, proactive control is considered to engage more cognitive resources than reactive control [20]. The concept of cognitive resources was previously linked to the constructs of working memory capacity and fluid intelligence (i.e., reasoning and problem solving abilities), and it was assumed that these two cognitive components could favor the ability to maintain goal-relevant information in an interfering context [13,24]. Thus it seems relevant to assume that individuals with high fluid intelligence should be more disposed to use proactive control to maintain task-goals in an active state in order to more efficiently manage the deleterious effect of interference. In accordance with this assumption, Perfetti, Tesse, Varanese, Saggino, and Onofrj [25] investigated the potential link between fluid intelligence and a cognitive bias occurring with task–irrelevant characteristics of a salient stimulus. In their study, Perfetti et al. [25] used a three-back working memory task in which continuous sequences of stimuli were presented to participants. Two versions of the task were used to assess “cross-domain biasing effects”. In the letter-detection version, participants were explicitly asked to retain and match the stimuli on the basis of their identity (letters), whereas they had to match stimuli according to their position for the spatial version of the task. Target and non-target trials were manipulated to introduce lures and create facilitating and interfering effects respectively. In other words, to measure “cross-domain biasing effects”, some trials involved a stimulus that matched the three-back items on the other task-domain than that requested for the response. As expected, Perfetti et al. [25] found that significant interference effects occurred for non-target stimuli following a three-back item matching the irrelevant domain. Moreover, they showed that fluid intelligence (assessed by the Raven’s Advanced Progressive Matrices [26]) could influence the ability to manage these interference effects across distinct domains. The authors suggested that the explicit instruction given to participants to focus on the task-relevant domain (letter-detection vs. spatial task) might have induced the use of proactive control strategy, allowing to improve goal representations maintenance in working memory to prevent interference effects, and this more particularly in the group with high fluid intelligence level.

In their 2010 study, Burgess and Braver [7] observed that young adults with high fluid intelligence levels were less sensitive to interference than low-level participants. However, this decreased interference effect was limited to the high-interference condition, in which proactive control is thought to be involved. Thus, this study also provided some evidence supporting the notion that individuals with high fluid intelligence levels tend to increase the use of the proactive control strategy in high-interference conditions.

#### Processing speed and cognitive control

Despite the absence of studies that directly investigate the impact of processing speed on cognitive control efficiency, several authors have suggested that there exists a relationship between fluid intelligence and processing speed [27-31], and also with working memory (for a review, [32]). For instance, Jensen [33] suggested that individual differences in working memory could underlie the correlation between speed and intelligence. Indeed, as information needed for reasoning and problem solving is temporarily stored in working memory, faster processing speed allows the completion of these processes before the loss of this information, leading to better performance. Accordingly, working memory was found to be correlated with reasoning abilities and processing speed in a series of experiment [34].

These data clearly showed a relationship between individual differences in processing speed, working memory and fluid intelligence in young adults. Moreover, other studies have indicated that working memory [13,35,36] and fluid intelligence [7,24] could influence proactive control abilities. Consequently, it seems relevant to explore the potential impact of processing speed and/or fluid intelligence on the tendency to efficiently select the most appropriate control strategy to perform a task, and more generally, the impact of these variables (considered to reflect the amount of available cognitive resources) on the selective age-related decline suggested in proactive control.

#### Aging and cognitive control

Age-related cognitive changes appear particularly pronounced in tasks that require a high degree of cognitive control, such as when attention must be endogenously and intensively focused, especially in the face of distraction and interference, as well as in cognitive situations that demand a large amount of attentional resources for a review, see [37]. Accordingly, a series of studies from Braver and colleagues showed a selective age-related impairment in proactive control, whereas the use of a reactive control strategy seems to remain intact [14,38,39].

Changes in cognitive control strategy in healthy older adults were notably investigated with the AX-CPT task ([38]; see also [14,39]. The classical AX-CPT paradigm requires participants to respond as quickly and accurately as possible to a specific target occurring after a specific cue. Target trials, which constitute 70% of the task, involve the occurrence of an A letter as the cue followed by an X letter as the target and require a positive response. Three kinds of non-target trials (10% each, for a total of 30% of the task), which require negative responses, involve (1) an invalid cue (non-A letter) preceding the X target (BX trials); (2) a valid cue (A letter) followed by an invalid “non-X” target (AY trials); and (3) an invalid cue (non-A letter) followed by an invalid “non-X” target (BY trials). With this task, Braver et al. [38] observed that older adults were more accurate than younger ones in the AY condition but the two groups of participants did not differ for BX and BY trials. Moreover, older adults were less disturbed by the A probe in non-target trials (smaller difference in reaction times [RTs] between AY and BY trials than in the young group). In contrast, older adults showed greater interference effects for the X probe (larger difference in RTs between BX and BY trials). These results suggest that older adults showed impairments affecting context representations and updating inasmuch as they had poorer BX performance (slower RTs but intact accuracy) and better AY performance (greater accuracy) than younger adults. Given that reactive control is defined as a transient reactivation of context representations following the occurrence of the probe, it may be assumed that older adults tended to rely on this kind of cognitive control because of their high accuracy on BX trials, which suggests that their access to context representations is spared, and their slower RTs on BX trials, suggesting that these context representations were reactivated when the probe occurred. Therefore, Braver et al. [38] proposed that there is an age-related impairment in the tendency to use proactive control to correctly prepare attentional mechanisms to process upcoming probe stimuli. This assumption seems to be supported by the enhanced age-related performance on AY trials, which could be interpreted as an age-related decline in the use of context representations to anticipate the probe (proactive control).

Thus, empirical evidence reveals an age-related decline in proactive control abilities. However, to our knowledge, no study has directly tested, in the context of the DMC framework, the hypothesis that the age-related decrease in proactive control abilities could in fact be influenced by the existence of less efficient general cognitive processes (such as processing speed and fluid intelligence). Answering this question should improve our understanding of cognitive control and the variations occurring on these mechanisms with age.

### Objectives of the study

Given the dynamic relationship between cognitive control, fluid intelligence and processing speed, as well as the age-related changes in these cognitive domains, the objective of this study was to explore the possible impact of fluid intelligence level and processing speed on the decline of proactive control abilities in healthy aging. Using a modified Sternberg paradigm [23], proactive and reactive control abilities were compared in young and older participants using three approaches: (1) an initial large sample of participants; (2) a subsample of young and older participants matched for fluid intelligence level; and (3) another subsample of young and older participants matched for processing speed. We hypothesized that a decrease in proactive control abilities would be observed in the initial sample but that no difference in performance would emerge when the influence of any age-related decline in fluid intelligence and processing speed was controlled. In order to extend results from the first analyses, hierarchical linear regressions were performed on the whole sample of participants’ scores in the high interference condition, using age, fluid intelligence level and processing speed as predictive variables.

## Methods

### Participants

Eighty young (43 men; M age = 22.1 years; SD = 2.8; range = 18–29) and 80 healthy older adults (37 men; M = 74.1 years; SD = 7.8; range = 60–89) were included in this study. Informed written consent was obtained from each participant; the study was approved by the Ethics Committee of the Faculty of Psychology of the University of Liège, and was conducted in accordance with the ethical standards described in the Declaration of Helsinki (1964). All participants had normal or corrected vision and hearing, were native French speakers and none reported any medical, neurological or sensory defects, or use of medication likely to alter cognitive functioning. The cognitive status of the older participants was checked with the Mattis Dementia Rating Scale [40] (see Table 1). All older participants had a total score equal to or greater than 130 (range 130–144), which constitutes the cut-off score to distinguish between healthy aging and dementia [41]. The young and older groups of participants differed in terms of educational level (t (158) = −2.014; *p* < .05) and vocabulary level on the French adaptation of the Mill Hill test [42] (t (158) = −8.342; *p* < .001) (see Table 1).

**Table 1:** Demographic and cognitive information for the whole sample of participants

	**Young adults (n = 80)**	**Older adults (n = 80)**
Gender ratio [male/female]	43/37	37/43
Educational level [years completed]	13.57 ± 2.28	14.5 ± 3.42
Mill Hill [Crystallized intelligence]	21.3 ± 5.09	27.5 ± 4.27
Raven’s advanced progressive matrices [Fluid intelligence]	52.85 ± 5.25	43.96 ± 7.74
Code test (WAIS-III) [Processing speed]	85.23 ± 12.68	57.64 ± 12.99
Mattis dementia rating scale		140.262 ± 0.291

Mean raw score ± standard deviation.

### Materials and procedure

Participants were tested individually in a quiet, well-lit room. The two conditions of the probe recency Sternberg task were presented on a microcomputer with a 15-inch color monitor using E-Prime software version 1.1, Service pack 3 [43]. Fluid intelligence and processing speed were respectively assessed by the paper-pencil version of the Raven’s Advanced Standard Progressive Matrices [44] and the Code task from the Wechsler Adult Intelligence Scale (WAIS-III; [45]). For the probe recency task, participants were seated in front of the computer screen at approximately 50 cm from the display. Response keys were located on a standard AZERTY keyboard.

The order of task administration was counterbalanced across participants such that half of the participants performed the high-interference condition of the probe recency task before the low-interference condition and the other half performed first the low-interference condition. Student t-tests performed on RTs and accuracy scores in the high- and low-interference conditions revealed no significant difference between the two orders of task administration, neither in the young nor in the older adults group (all *p*s > .05).

### Tasks

#### Fluid intelligence

All participants performed the Raven’s Advanced Progressive Matrices [44] (see Table 1). In this 60-item non-verbal reasoning test, each item contains a pattern with a missing piece. The subject has to infer the rules underlying the pattern and apply these rules to discover which of the answer options provides the correct completion. Completion was self-paced. The score obtained is assumed to provide a reliable measure of reasoning and fluid intelligence level.

#### Processing speed

Participants also performed the Code test from the WAIS-III [45]. This task requires participants to write, as quickly and accurately as possible, the corresponding numbers in front of symbols with the help of a correspondence table (see Table 1). The score obtained is thought to reveal basic processing speed abilities.

#### The probe recency task

**Figure 1:**
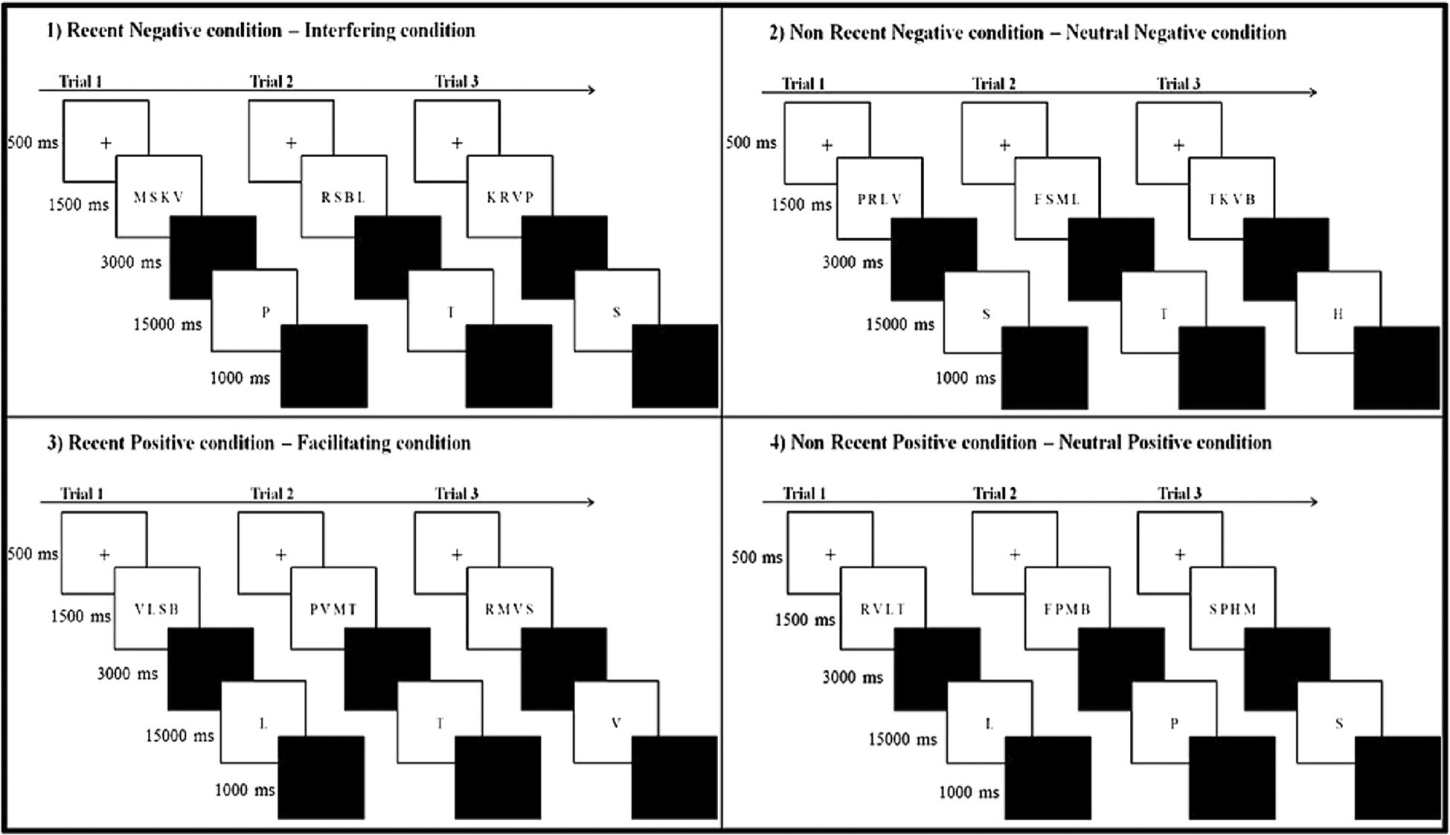
**Task conditions of the Sternberg paradigm.** The four task conditions determined by the nature of the probe items. (1) Recent negative condition: The probe did not match any items of the current target set but had occurred in both previous trials; (2) Non-recent negative condition: The probe did not match any items of the current target set or of the two previous trials; (3) Recent positive condition: The probe matched an item of the current target set and had occurred in both previous trials; (4) Non-recent positive condition: The probe matched an item of the current target set but had not occurred in the two previous trials.

Cognitive control was assessed by an adapted Sternberg’s [23] item-recognition short-term memory task. Participants were presented series of trials consisting in groups of four consonants (target groups). They had to maintain these items in memory for a short retention interval, after which they were given a single probe item and had to decide whether this probe matched one of the items previously presented in the target group. The time course of a trial was as follows (see Figure 1): First, a fixation cross was displayed for 500 ms, followed by the visual presentation of the group of four consonants for 1500 ms. A blank screen was then displayed for 3000 ms, followed by the probe letter. The probe letter remained on the screen until the response was given, with a maximum response time allowed of 15 s. Finally, a blank screen was presented again for 1000 ms before the beginning of the next trial. Participants had to indicate, as quickly and accurately as possible, by pressing one of two response keys, whether the probe letter was present or not in the four-letter target group of the current trial.

There were four trial types (illustrated in Figure 1) defined by the nature of the probe: (1) *recent negative trials*, for which the probe did not match any items from the current target set (and thus required a “no” response), but did match an item from the two previous target sets; (2) *non-recent negative trials*, for which the probe did not match any item from the current target set (and thus required a “no” response) nor the two previous target sets; (3) *recent positive trials*, for which the probe matched an item that was presented in the current target set (and thus required a “yes” response) and also in the two previous target sets; (4) *non-recent positive trials*, for which the probe matched an item that was presented in the current target set (and thus required a “yes” response) but not in the two previous target sets. Recent negative trials constituted the *interfering trials*, and recent positive trials the *facilitating trials*. Non-recent negative and positive trials represent control trials, used to calculate interference and facilitation effects respectively (see below). In the present study, only interference effects will be discussed.

To manipulate the recruitment of proactive and reactive control processes, two versions of the probe recency task were created by varying the ratio of recent positive and recent negative trials in each version (see Table 2 for items distribution). Indeed, according to Braver, Gray, & Burgess [20], a high probability of encountering interfering items (here, recent negative items) across trials should favor the employment of a proactive control strategy, whereas a low probability of interference should favor reactive control. Consequently, the low-interference condition was composed of 40% recent positive trials and 10% recent negative trials while the reverse proportion (40% recent negative and 10% recent positive) was presented in the high-interference condition. The proportion of non-recent positive and non-recent negative (neutral items) was 25% in each condition.

**Table 2:** Composition of the low- and the high-interference conditions

**Nature of probes**	**Low-interference condition**	**High-interference condition**
	**[Reactive Control]**	**[Proactive Control]**
Recent positive	40% (32)	10% (8)
Recent negative	10% (8)	40% (32)
Non-recent positive	25% (20)	25% (20)
Non-recent negative	25% (20)	25% (20)

Distribution of the probe items in the two parts of the probe recency task (Raw number of trials).

## Results

All analyses were conducted with a statistical threshold set at *p* < .05. In order to meet the assumptions of homogeneity of variances and normality, statistical analyses were performed on logarithmic transformed RTs and arcsine transformed accuracy scores. Nevertheless, for the sake of clarity, figures were created using the means of the raw values. An interference index was calculated by subtracting RTs for non-recent negative stimuli (“neutral stimuli”) from recent negative stimuli (“interfering stimuli”) in both high- and low-interference conditions. With regard to accuracy, the reverse interference index (non-recent negative – recent negative) was calculated. Consequently, high scores are indicative of considerable sensitivity to interference for both RTs and accuracy.

First, in order to determine the age-related effect on proactive and reactive cognitive control processes, two repeated measures ANOVAs were performed on RTs and accuracy scores comparing the two groups of participants, with task condition (high or low interference level) as repeated measure factor. Planned contrasts were used to test the effect of age in proactive and reactive control. Older adults were expected to have more difficulties to manage interference in the high-interference condition (reflecting proactive control) than younger adults. However, no significant difference was expected between the two groups in the condition thought to favor the use of reactive control strategy (low-interference condition). In addition, given that a significant difference in educational level was evidenced between young and older adults, the same repeated measures ANOVAs were conducted including educational level as covariate.

To investigate the potential influence of age-related differences in cognitive resources on the selective age-related decline in proactive control, two statistical approaches were used. First, repeated measures ANOVAs were conducted on subgroups within the participant sample that included young and older adults who were matched on the basis of their performance on fluid intelligence and processing speed tasks, respectively. Score ranges were defined to create the largest subgroups of young and older participants demonstrating similar performance in both fluid intelligence and processing speed to avoid significant differences between subsamples. With regard to the effect of fluid intelligence level, 25 young adults and 25 older adults with Raven scores between 48 and 53 were included. Similarly, for processing speed, 25 young adults (Code score between 61 and 89) and 29 older adults (Code score between 61 and 93) were considered. The use of matched groups for fluid intelligence abilities on one hand and for processing speed on the other hand should provide a first evidence of the potential impact of cognitive resources on the postulated specific age-related decline in proactive control. Indeed, if the tendency to use proactive control to deal with interference in high-interference conditions is only sensitive to aging, differences should persist in paired participants. However, if between-group differences disappeared, a potential impact of cognitive resources might be suspected. Again, planned comparisons were conducted between paired subgroups in the high- and low-interference conditions to observe the potential selectivity of the results in the high-interference condition (reflecting proactive control). The effect size of each repeated measures ANOVA was reported as eta squared (*η*^*2*^) for the main effects and interactions. Eta squared is generally interpreted as the proportion of variance of the dependent variable that is related to the factor. Traditionally, values of .01, .06, and .14 represent small, medium and large effect sizes, respectively [46]. For planned comparisons, the effect size was reported using Cohen’s D (*d*) for which values of .2, .5, and .8 represent small, medium and large effects, respectively.

Second, the privileged relationship that could exist between proactive control and cognitive resources was more directly investigated in the whole sample of participants with hierarchical linear regression analyses on the performance in the high-interference condition. Fluid intelligence level and processing speed were considered as a “block” of cognitive resources and were included simultaneously in the analyses. In a first step, hierarchical linear regressions were conducted to assess whether the age-related variance in interference sensitivity in the high-interference condition remain significant after partialling out the percentage of variance explained by cognitive resources. Afterwards, hierarchical regression models were constructed to measure variance of performance in the high-interference condition that might be explained by cognitive resources after controlling for age-related variance.

### Selective age-related decline in proactive control

While only interference effects were reported in the present work, for the sake of completeness, raw performance on the whole task was reported in Table 3.

**Table 3:** Young and older adults groups’ performance on the probe recency task

	**Young adults (n = 80)**		**Older adults (n = 80)**	
	**High-interference condition**	**Low-interference condition**	**High-interference condition**	**Low-interference condition**
***Recent negative***				
*RTs*	904.337 ± 212.009	879.987 ± 231.631	1310.519 ± 346.285	1262.706 ± 317.558
*Accuracy*	0.933 ± 0.074	0.972 ± 0.069	0.912 ± 0.103	0.969 ± 0.083
***Non-recent negative***				
*RTs*	777.925 ± 162.656	796.506 ± 174.007	1108.05 ± 276.049	1144.569 ± 264.464
*Accuracy*	0.987 ± 0.027	0.982 ± 0.032	0.976 ± 0.05	0.974 ± 0.039
***Recent positive***				
*RTs*	784.231 ± 153.083	781.775 ± 143.168	1136.431 ± 314.061	1084.181 ± 230.038
*Accuracy*	0.959 ± 0.074	0.957 ± 0.047	0.948 ± 0.091	0.941 ± 0.06
***Non-recent positive***				
*RTs*	809.244 ± 147.426	799.05 ± 157.734	1150.2 ± 280.182	1121.537 ± 246.568
*Accuracy*	0.911 ± 0.089	0.915 ± 0.086	0.886 ± 0.09	0.896 ± 0.091

Mean raw median RTs ± standard deviations.

**Figure 2:**
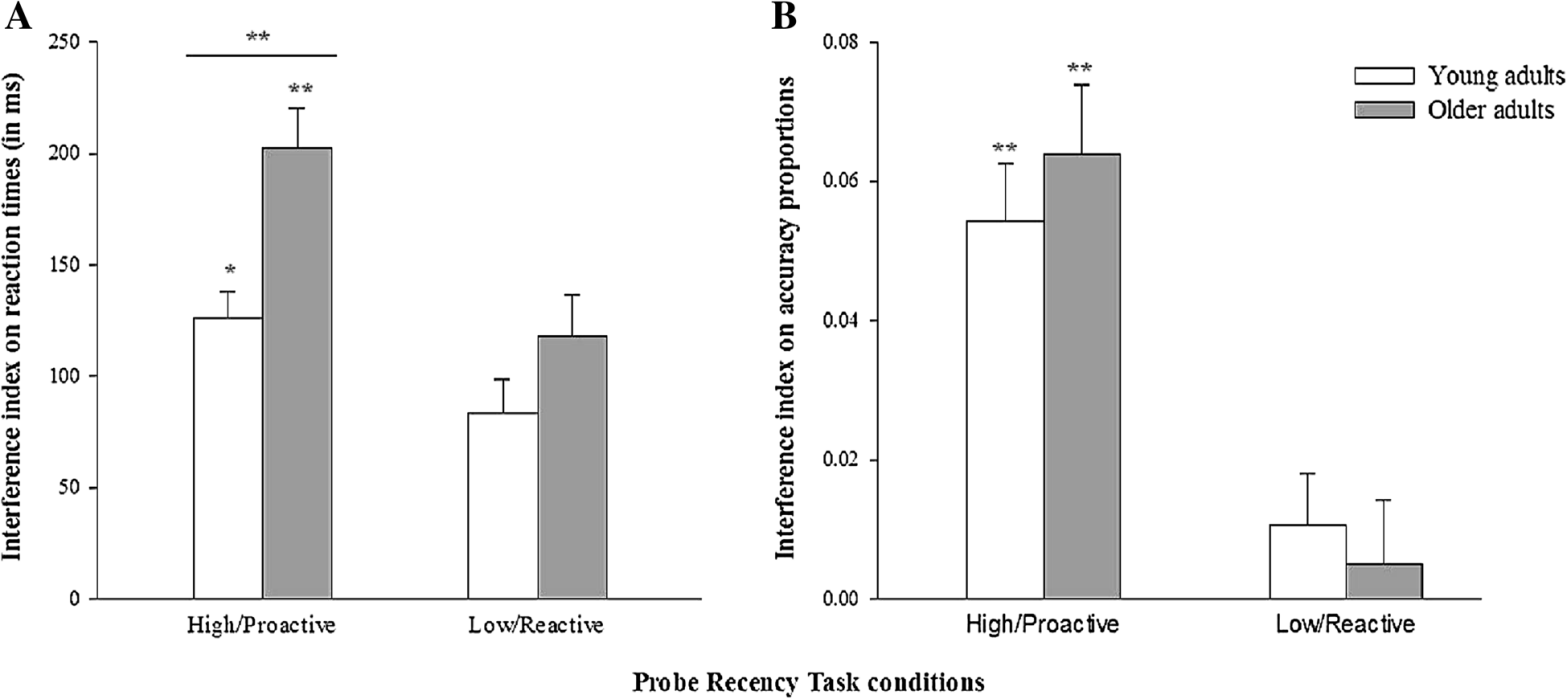
**Interference sensitivity in high (proactive) and low (reactive) interference conditions for young vs. older adults. (A)** Mean reaction times (ms); **(B)** Accuracy proportions. Error bars represent standard errors. *p < .05; **p < .001.

A 2 (group: young vs. older adults) × 2 (condition: high vs. low interference level) repeated measures ANOVA was performed on RTs (Figure 2A), and revealed significant effects of age (F(1,158) = 8.3; *p* < .01; *η*^*2*^ = 0.05), with greater sensitivity to interference in the older group; condition (F(1,158) = 25.3; *p* < .001; *η*^*2*^ = 0.138), with smaller interference indices in the low-interference condition; but no significant age*condition interaction (F(1,158) = 2.1; *p* = .149; *η*^*2*^ = 0.013). Planned comparisons revealed greater sensitivity to interference for older than younger adults in the high-interference condition (F(1,158) = 11.683; *p* < .001; *d* = 0.54), while no group difference was found in the low-interference condition (F(1,158) = 2.144; *p* = .145; *d* = 0.22).

Concerning accuracy (Figure 2B), the 2 (group: young vs. older adults) × 2 (condition: high vs. low interference level) repeated measures ANOVA revealed only a significant effect of condition (F(1,158) = 76.914; *p* < .001; *η*^*2*^ = 0.33), with smaller interference indices in the low-interference condition; but no significant age effect (F(1,158) = 0.21; *p* = .651; *η*^*2*^ = 0.001) or age*condition interaction (F(1,158) = 0.61; *p* = .436; *η*^*2*^ = 0.004). Planned comparisons between young and older adults confirmed the absence of age-related interference sensitivity in high (proactive) (F(1,158) = 0.039; *p* = .844; *d* = 0.031) and low (reactive) (F(1,158) = 0.792; *p* = .375; *d* = 0.142) interference conditions.

Finally, two 2 (group: young vs. older adults) × 2 (condition: high vs. low interference level) repeated measures ANCOVAs were conducted with years of education as covariate to discard the potential influence of educational level on RTs and accuracy performance. Concerning RTs, no significant effect of the educational level was found (F(1,157) = 0.00; *p* = .97; *η*^*2*^ < 0.001), the effect of age (F(1,157) = 7.99; *p < .05; η*^*2*^*= 0.05)* remained significant and the age* condition interaction (F(1,157) = 2.48; *p* = .12; *η*^*2*^ = 0.02) remained non-significant, while the effect of condition disappeared (F(1,157) = 3.67; *p* = .057; *η*^*2*^ = 0.02). With regard to accuracy data, the analysis revealed a significant effect of educational level (F(1,157) = 8.37; *p < .*05; *η*^*2*^ = 0.05). The other effects were not modified by adding the covariate: the age effect remained non-significant (F(1,157) = 0.00; *p* = .99; *η*^*2*^ = 0), the condition effect significant (F(1,157) = 17.31; *p* < .001; *η*^*2*^ = 0.1) and the age*condition interaction non-significant (F(1,157) = 1.36; *p* = .25; *η*^*2*^ = 0.01).

In sum, the analyses of RTs revealed a selective age-related decline in proactive control (high-interference condition) whereas reactive control abilities seem to be preserved. However, accuracy analyses evidenced a very small number of errors in both high- and low-interference conditions and did not show any effect of age on interference sensitivity. Therefore, to improve our understanding of cognitive control mechanisms and the effects of age on these processes, it seems relevant to explore whether fluid intelligence level and processing speed might influence this age-related decrease in continuous management of interference. Due to the absence of an age-related decline in cognitive control in terms of accuracy, only RTs were considered in the subsequent analyses.

### Impact of fluid intelligence and processing speed on cognitive control abilities in healthy aging

Concerning fluid intelligence level, a Student *t* test performed on the entire participant sample (n = 160) revealed a significant difference between young and older adults performance on Raven’s Progressive Matrices (t(158) = 8.721; *p* < .001), with the younger group performing better (see Table 1), which confirms the presence of an age-related decline in fluid intelligence. To examine the potential influence of fluid intelligence on proactive control abilities in aging, subgroups were formed within the participant sample. As described above, 25 young (*M* age = 21.64; SD = 3.289) and 25 older adults (*M* age = 73.8; SD = 7.047) with scores between 48 and 53 on the Raven’s Advanced Progressive Matrices test were selected (see Table 4). As expected, the performance of these subsamples of young and older participants was similar (t(48) = 0.606; *p* = .547).

**Table 4:** Demographic data and cognitive assessment in subgroups of participants matched for fluid intelligence and processing speed

		**Gender ratio [Male/female]**	**Age**	**Educational level [Years completed]**	**Mill hill [Crystallized intelligence]**	**Raven’s advanced progressive matrices [Fluid intelligence]**	**Code test (WAIS-III) [Processing speed]**	**Mattis dementia rating scale**
**Fluid intelligence matching**	Young adults (n = 25)	14/11	21.64 ± 3.29	12.72 ± 2.07	19.56 ± 5.07	50.6 ± 1.78	79.52 ± 9.46	
	Older adults (n = 25)	13/12	73.8 ± 7.05	15.04 ± 3.67	29.56 ± 2.52	50.32 ± 1.6	63.56 ± 9.93	141.92 ± 2.29
	Student t tests between young and older adults			t(48) = −2.753*	t(48) = −8.998**	**t(48) = 0.606**	t(48) = 5.775**	
**Processing speed matching**	Young adults (n = 25)	14/11	21.96 ± 2.41	13.08 ± 2.39	20.52 ± 4.96	50.12 ± 5.44	74.64 ± 7.47	
	Older adults (n = 29)	12/17	70.72 ± 7.64	14.10 ± 2.69	28.86 ± 3.04	45.97 ± 7.81	71.07 ± 8.15	141.79 ± 2.31
	Student t tests between young and older adults			t(52) = −1.465	t(52) = −7.777**	t(52) = 2.208*	**t(52) = 1.651**	

Mean raw scores ± standard deviations *p <.05; ** *p* <.001.

Student t tests reported in boldface shows no significant difference in fluid intelligence (*p*=.581)or in processing speed (*p*=.662) between subgroups of young and older adults that were respectively paired for these cognitive resources.

Concerning processing speed, a Student *t* test conducted on the whole participant sample revealed a significant difference between young and older adults (t(158) = 13.322; *p* < .001). As Table 1 shows, young adults performed significantly better than older adults on the Code task, confirming the postulated age-related decline in processing speed. To determine the influence of processing speed on the age-related decline in proactive control abilities, two subgroups of 25 young adults with a score on the Code test between 61 and 89 (*M* age = 21.96; SD = 2.406) and 29 older adults with a score between 61 and 93 (*M* age = 70.724; SD = 7.643) were created (see Table 4). Again, the difference in performance between these subgroups became non-significant (t(52) = 1.651; *p* = .105).

Separate ANOVAs were performed on the subgroups matched for fluid intelligence level and processing speed. Due to the similar level of performance between young and older participants on these factors, the analyses were conducted to investigate whether the proactive control decline evidenced in our first analysis can be considered as a specific effect of aging or influenced by a decrease in fluid intelligence and/or processing speed. Indeed, in the case of a specific effect of aging, the age-related difference in proactive control should remain significant. However, this difference was expected to disappear if it was influenced by a decrease in fluid intelligence and/or processing speed.

### Influence of fluid intelligence on the age-related decline in proactive control

**Figure 3:**
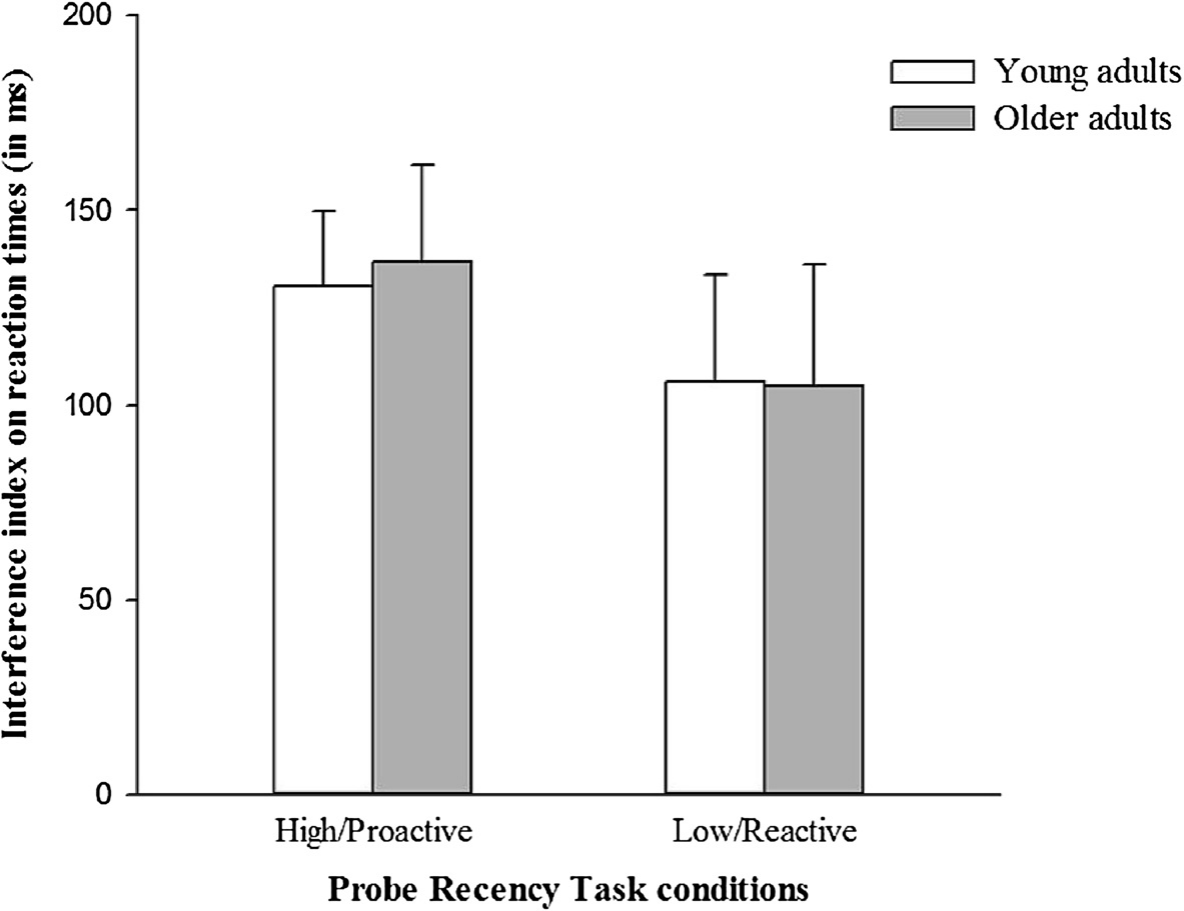
**Interference sensitivity in high (proactive) and low (reactive) interference conditions for young vs. older adults with similar fluid intelligence level.** Mean reaction times (ms); Error bars represent standard errors. *p < .05; **p < .001.

A 2 (paired subgroup: young vs. older adults) × 2 (condition: high vs. low interference level) repeated measures ANOVA was performed to investigate the potential influence of fluid intelligence on the selective age-related decline in proactive control (Table 5 and Figure 3). The analysis of RTs revealed no significant effect of age (F(1,48) = 0.00; *p* = .957; *η*^*2*^ = 0.00006), suggesting no significant difference in interference sensitivity between young and older adults who have similar fluid intelligence level. No significant effect of condition was found (F(1,48) = 2.87; *p* = .097; *η*^*2*^ = 0.056), suggesting no significant difference in interference sensitivity between high and low-interference conditions. Finally, no significant age*condition interaction (F(1,48) = 0.03; *p* = .861; *η*^*2*^ = 0.0006) was revealed, suggesting that young and older adults did not differ in sensitivity to interference in proactive and reactive control conditions. These results were confirmed by planned comparisons that showed no significant difference in interference sensitivity in the two groups both in the high (F(1,48) = 0.005; *p* = .944; *d* = 0.014) and low (F(1,48) = 0.017; *p* = .897; *d* = 0.041) interference conditions.

**Table 5:** Probe Recency Task performance (RTs) for fluid intelligence subgroups

	***Young adults (n = 25)***		***Older adults (n = 25)***	
	***High-interference condition***	***Low-interference condition***	***High-interference condition***	***Low-interference condition***
*Recent negative*	950.02 ± 206.812	946.48 ± 226.763	1145.5 ± 204.028	1151.4 ± 266.354
*Non-recent negative*	819.18 ± 173.024	840.44 ± 192.809	1008.7 ± 171.459	1046.36 ± 202.262
*Recent positive*	833.5 ± 171.849	803.64 ± 135.751	1076.56 ± 269.702	991.66 ± 156.991
*Non-recent positive*	849.56 ± 170.973	814.86 ± 161.804	1070.74 ± 160.817	1035.88 ± 217.716

Mean raw median RTs ± standard deviations.

### Influence of processing speed on the age-related decline in proactive control

**Figure 4:**
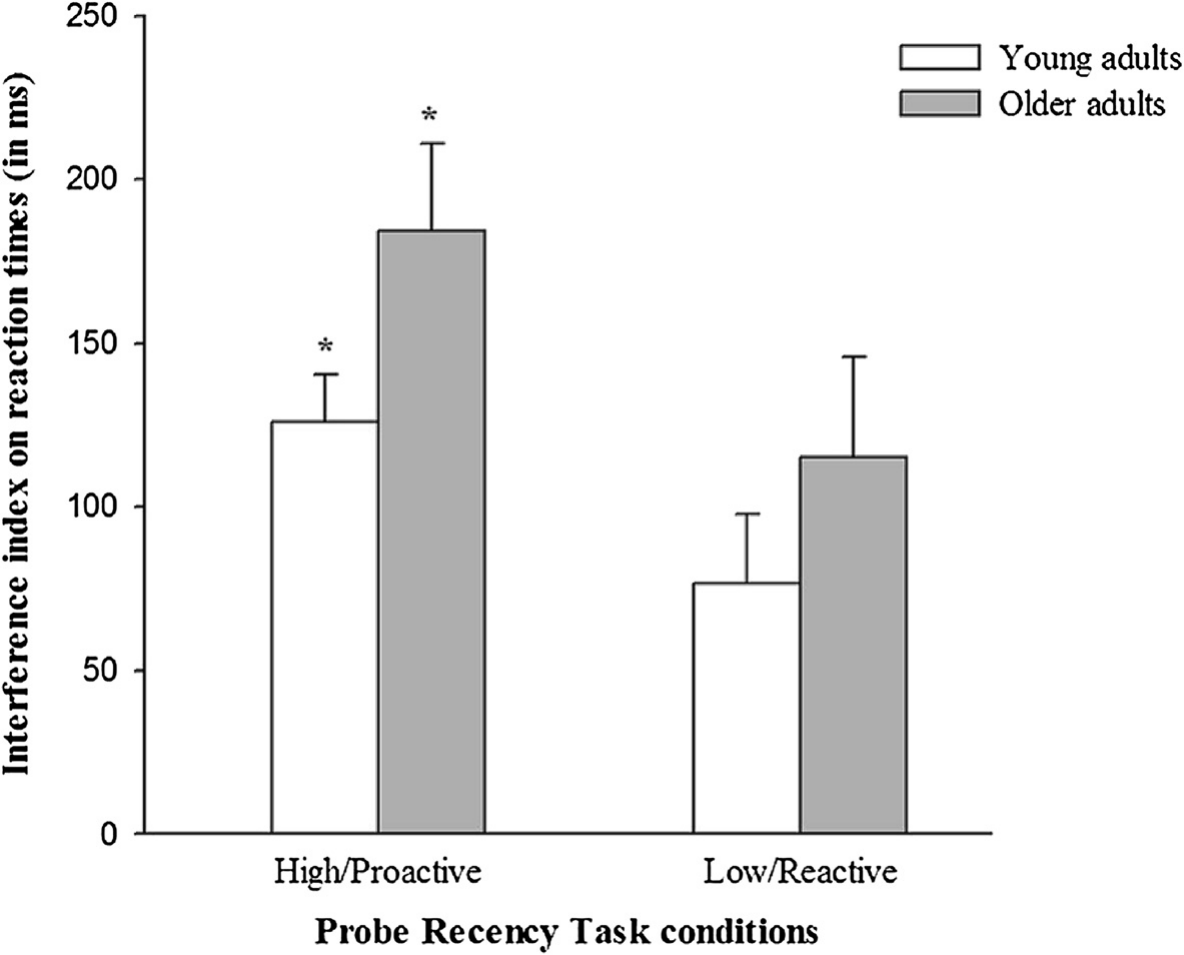
**Interference sensitivity in high (proactive) and low (reactive) interference conditions for young vs. older adults with similar processing speed.** Mean reaction times (ms); Error bars represent standard errors**.** *p < .05; **p < .001.

A 2 (paired subgroup: young vs. older adults) × 2 (condition: high vs. low interference level) repeated measures ANOVA was performed to investigate the potential influence of processing speed on the selective age-related decline in proactive control (Table 6 and Figure 4). The RTs analysis revealed no significant effect of age (F(1,52) = 2.11; *p* = .152; *η*^*2*^ = 0.039), suggesting no significant difference in interference sensitivity between young and older adults. A significant effect of condition (F(1,52) = 11.66; *p* < .05; *η*^*2*^ = 0.183), with smaller interference indices in the low-interference condition, was found. Finally, no significant age*condition interaction (F(1,52) = 0.17; *p* = .686; *η*^*2*^ = 0.003) was evidenced, suggesting that young and older adults did not differ in interference sensitivity across the two conditions (high- and low-interference). Moreover, planned comparisons confirmed that young and older adults did not differ in interference sensitivity in both high (F(1,52) = 2.544; *p* = .117; *d* = 0.438) and low (F(1,52) = 0.756; *p* = .388; *d* = 0.243) interference conditions.

**Table 6:** Probe Recency Task performance (RTs) for processing speed subgroups

	***Young adults (n = 25)***		***Older adults (n = 29)***	
	***High-Interference condition***	***Low-Interference condition***	***High-Interference condition***	***Low-Interference condition***
*Recent negative*	907.28 ± 184.188	874.64 ± 206.275	1168.724 ± 263.362	1130.138 ± 294.32
*Non-recent negative*	781.08 ± 145.053	798.18 ± 160.469	984.293 ± 204.173	1015.069 ± 220.5
*Recent positive*	829.28 ± 194.105	792.22 ± 141.983	1020.069 ± 241.048	968.466 ± 193.416
*Non-recent positive*	845.32 ± 154.266	810.7 ± 147.945	1030.552 ± 184.817	1014.862 ± 235.801

Mean raw median RTs ± standard deviations.

### Hierarchical linear regressions

In order to more directly test the influence of cognitive resources (fluid intelligence and processing speed) on the observed age-related decline in proactive control, two hierarchical linear regression analyses were performed on RTs in the high-interference condition. For these analyses, fluid intelligence and processing speed performance were not collapsed in a composite score because they were only moderately correlated (r < .70 as preconized by Hair, Black, Babin, & Anderson [47]) (r = .48 in the young adults group and r = .39 in the older adults group), but were simultaneously entered in the same block of predictors to assess the “cognitive resources” influence on age-related proactive control performance.

First, hierarchical regressions were conducted to assess whether the age-related variance in interference sensitivity in the high-interference condition remain significant after controlling for variance explained by cognitive resources (Models A). Results are summarized in Table 7A. The model 1A provides a simple linear regression assessing the amount of variance in the high-interference condition that could be attributed to age. In order to verify whether the contribution of age might be reduced to a non-significant account after controlling for cognitive resources-related variance in the high-interference condition, the model 2A includes fluid intelligence and processing speed performance as cognitive resources predictor.

**Table 7:** Results from hierarchical linear regressions

										
**A**	***Model***	***Step***	***Predictors***	***R***^***2***^	***Adjusted R***^***2***^	***SE***	***ΔR***^***2***^	***ΔF***	***p***	***β***
	1. A	1	Age groups	0.069	0.063	0.049	0.069	11.684	0.001	0.262
	2. A	1	Fluid intelligence & processing speed	0.097	0.086	0.048	0.097	8.478	0.000	−0.247
										0.03
		2	Age groups	0.107	0.09	0.048	0.009	1.638	0.202	0.143
**B**	***Model***	***Step***	***Predictors***	***R***^***2***^	***Adjusted R***^***2***^	***SE***	***ΔR***^***2***^	***ΔF***	***p***	***β***
	1. B	1	Fluid intelligence & processing speed	0.097	0.086	0.048	0.097	8.478	0.000	−0.27
										−0.059
	2. B	1	Age groups	0.069	0.063	0.049	0.069	11.684	0.001	0.143
		2	Fluid intelligence & processing speed	0.107	0.09	0.048	0.038	3.319	0.039	−0.247
										0.03

**A)** Results from the hierarchical linear regressions performed on the high-interference condition to investigate the age-related influence on interference sensitivity after controlling for cognitive resources. **B)** Results from the hierarchical linear regressions performed on the high-interference condition to investigate the cognitive resources-related influence on interference sensitivity after controlling for age.

Model 1A revealed that age significantly explained 6.9% of the variance in the high-interference condition (F(1,158) = 11.684; *p* < .001). In the second model (2A), the age-related variance became non significant (ΔF(1,156) = 1.638; *p* = .202) after controlling for cognitive resources (ΔF(2,157) = 8.478; *p <* 0.001). Following Baudouin et al.’s procedure^a^[48], cognitive resources accounted for 86.95% of the age-related variance of interference management in the high-interference condition.

Second, hierarchical regression models were constructed to assess variance of performance in the high-interference condition that might be explained by cognitive resources after controlling for age-related influence (Models B). Results are summarized in Table 7B. The first model (1B), providing a simple linear regression to assess the amount of variance in the high-interference condition that could be attributed to cognitive resources, revealed a significant explained variance of 9.7% (F(2,157) = 8.478; *p* < .001). In order to verify whether the contribution of cognitive resources might be reduced to a non-significant account after controlling for age-related variance in the high demanding condition, the model 2B was constructed using age groups as first predictor and cognitive resources as second predictor. This second model confirmed that cognitive resources (ΔF(2,156) = 3.319; *p* < .05) added a significant explained variance to the interference sensitivity in the high-interference condition, once age-related variance (ΔF(1,158) = 11.684; *p* < .01) had been controlled. Finally, following the same procedure as before [48], the age groups accounted for 60.82% of the cognitive resources variance.

## Discussion

This study was designed to investigate, in the context of the DMC model [20], the age-related decline in cognitive control and particularly the decreased tendency to use proactive control strategies in high-interference conditions with aging. More specifically, we were interested in determining whether performance on general cognitive abilities such as fluid intelligence and processing speed might influence the selective age-related decline in proactive control.

The present study replicates previous findings [14,38,39] indicative of a selective age-related decline in proactive control, in association with preserved reactive control abilities. Indeed, greater sensitivity to interference was observed for older than younger adults in the high-interference condition of the Sternberg task, which is thought to favor proactive control, but not in the low-interference condition, which is postulated to involve reactive control processes. Therefore, the presence of greater sensitivity to interference (in comparison to young subjects) in a context involving a large number of interfering trials seems to indicate that older adults did not try to anticipate the occurrence of interfering items but rather tended to react following the presentation of these items [[14,38,39], for similar results and interpretation].

Conflict monitoring [11] and executive attention [13] theories stress the importance of active maintenance of goal-relevant information to achieve a task, particularly during high-interference situations. In line with these theories, the observed selective impairment of proactive control processes might be interpreted as a failure to maintain task-goal representations in a high-interference context see also [39] or, more generally, as a deficit affecting general context representations, including task-relevant information that could influence cognitive processes involved in the task [14]. In addition, similarly to the study conducted by Bélanger, Belleville and Gauthier [3], our results revealed an increase in age-related interference sensitivity in high-interference situations but particularly for RTs and not for accuracy of responses. Therefore, age seems to partially affect the ability to maintain task-goal information, since older adults were slowed down by the goal-maintenance requirement but they were still able to succeed on the trials.

In addition, one important goal of this study was to determine whether the decrease in proactive control abilities in healthy aging is related to fluid intelligence level and processing speed, two general cognitive processes known to be affected by age e.g., [49-54]. For instance, Li et al. [51] observed an age-related decline in fluid intelligence (composite score derived from adapted psychometric tasks used in the Berlin Aging Study [50,55]) from 40 years old. Decreased ability to efficiently manage conflict costs was also observed from the age of 50 using a flanker task. Interestingly, these authors also revealed a significant relationship between fluid intelligence and conflict costs for their older adults group while no significant influence appeared for younger adults, suggesting that the decline in cognitive resources abilities could impede the tendency to implement the most efficient strategy to perform a task.

Moreover, within the DMC background, the tendency to use proactive control was previously related to the ease or efficacy of active goal maintenance in working memory [13,21,56]. Thus, due to the interrelations between working memory and fluid intelligence/processing speed [27,29,49,57], it seemed relevant to predict an influence of these cognitive resources on proactive control abilities. With regard to fluid intelligence, Burgess and Braver [7] observed that participants with high fluid intelligence were less sensitive to interference than participants with low fluid intelligence in the high-interference condition, in which proactive control is assumed to be involved. With the same kind of task, results obtained in the present study suggest that, when older and younger adults are equated for their level of fluid intelligence, the age effect on the use of proactive control in high-interference condition disappeared. Therefore, the current results are consistent with Burgess and Braver’s [7] earlier findings and extend the evidence to healthy aging, suggesting that fluid intelligence could influence how proactive and reactive cognitive control processes are implemented according to task requirements in both young and older adults.

Given the age-related decline in processing speed [58] and its influence on working memory abilities [53,59], this study also investigated the potential influence of this factor on the age-related decline in proactive control. Similarly to fluid intelligence, processing speed seems to be involved in the tendency to anticipate in a sustained manner the interfering items in a high-interference context. Indeed, when younger and older participants were selected according to their performance on a basic processing speed task, the previously observed selective age-related difference in proactive control disappeared.

In addition, hierarchical linear regression analyses were conducted on RTs to more directly assess the influence of cognitive resources on proactive control abilities. These analyses supported the assumption of an influence of age-related decline in cognitive resources (assessed here simultaneously by fluid intelligence and processing speed) on the tendency to use proactive control strategies in a high-interference condition. Indeed, the results of a first analysis suggest that age explains a significant amount of interference sensitivity variance in the high-interference condition, assumed to favor proactive control. Moreover, this analysis also indicates that cognitive resources account for a large portion of that age-related variance in proactive control. Finally, models including age groups in a first step, followed by cognitive resources, suggested that cognitive resources add explained variance to interference sensitivity in the high-interference condition, which is not captured by age-related variance.

### Limitations

It should be noted that the present study provided an unexpected effect of condition. Indeed, contrary to the prediction of Braver, Gray and Burgess [20], the high-interference condition seems more difficult for young and older participants. Even if the reason is not clear, since few studies have used the Sternberg paradigm to explore proactive and reactive control processes, this unexpected pattern of results should not impede the exploration of age-related changes in cognitive control because we systematically compared the performance of young and older participants in each condition separately. It seems interesting to note that this pattern of response was not influenced by a more liberal response bias in the high- than in the low- interference condition. Indeed, C indices were calculated according to the signal detection theory [60] and revealed a significant tendency for “yes” responses that did not differ across age-groups or across control conditions. Moreover, participants that did not show the expected effect in the high-interference condition did not differ on demographic (years of education) or on neuropsychological variables (fluid intelligence, crystallized intelligence, or processing speed). Therefore, cognitive resources factors that may influence cognitive control need further investigation if we want to better understand the variability observed in healthy populations.

Brain imaging data could be very valuable in investigating the implementation of proactive and reactive control strategies in healthy aging according to the availability of cognitive resources. For instance, the DMC model [20] distinguished two specific patterns of cerebral activity depending on the use of proactive or reactive control strategy. Proactive control was associated with phasic activity in the lateral prefrontal cortex, whereas reactive control involved transient activation, particularly in the anterior cingulate cortex, lateral prefrontal cortex and medial temporal lobe. In accordance with these predictions, Burgess and Braver [7] observed in young subjects transient increased activity in the lateral prefrontal cortex during the probe period when interference expectancy was low; but sustained bilateral prefrontal cortex activity during the delay period in situations of high expectation of interference. Moreover, the high fluid intelligence group demonstrated increased activation of right lateral prefrontal regions prior to the presentation of the probe (suggesting a proactive control strategy) compared to the low fluid intelligence group, which seemed to preferentially implement reactive control strategies (i.e., probe-triggered activation on interference trials). In addition, Braver et al. [20] proposed that the observed shift from proactive control to reactive control in aging was also supported by changes in brain activation and neurotransmission patterns, particularly in the prefrontal cortex and in the dopamine system, respectively. Therefore, it seems relevant to hypothesize that age-related prefrontal atrophy and/or intra-cerebral dopamine levels decrease might influence the efficiency in implementing proactive control processes leading to a pattern of brain activity reflecting the involvement of reactive control [see [39] for such a pattern of results using the AX-CPT task].

At this time, few studies explored the neural substrates of cognitive control in young subjects [7,61-64] and these studies used various protocols (e.g., Sternberg, Stroop and N-back tasks). Some discrepancies in the neural substrates associated to proactive and reactive control were observed according to the exact characteristics of the tasks (e.g. more or less initial requirement of reactive vs. proactive control) and explored populations (e.g., varying by the level of dopamine availability). However, the adaptation of these paradigms to aging populations should add a substantial value to the comprehension of the implementation of proactive and reactive control processes, as well as to the influence of available cognitive resources. In particular, such studies would improve the understanding of the cognitive factors that could exert an influence on the selective age-related decline in the ability to use proactive control in a high demanding environment.

## Conclusions

In conclusion, this study suggests that the age-related decline in the tendency to use efficiently the proactive control strategy is at least partially modulated by fluid intelligence level and processing speed abilities. Consequently, the ability to appropriately use cognitive control processes during aging seems to be related to the amount of available cognitive resources. Therefore, it could be suggested that limiting the decline in fluid intelligence and/or processing speed could improve the ability of healthy older adults to optimally cope with interference in high-demanding situations. For example, future studies could evaluate the potential benefit of specific cognitive resources training programs on the effective use of proactive control abilities in aging. Another interesting line of research could be the investigation of personality factors in older adults that might influence the tendency to adapt strategies according to the task context e.g. [65,66]. More generally, the present study emphasizes the importance of taking into account available cognitive resources during cognitive control assessment in healthy as well as pathological populations (e.g. schizophrenia or neurodegenerative diseases).

## Endnote

^a^According to these authors [48], the percentage of cognitive resources – related variance that accounted for the age effect can be calculated by the formulae: [ΔR^2^regressor from model 1 (simple model) – ΔR^2^regressor from model 2 (hierarchical model)/(ΔR^2^regressor from model 1)*100].

## Competing interests

The authors report no conflict of interest. The funders had no role in study design, data collection and analysis, decision to publish, or preparation of the manuscript.

## Authors’ contributions

FC conceived the study, was the first coordinator, provided substantial support to proofread the manuscript and gave the final approval of the version to be published. MJ participated in the study design and statistical analyses. DC realized the acquisition of data and participated in the material building. MM carried out statistical analyses and interpretations and was involved in the preparation of the manuscript. Intellectual content was collectively revised by all the authors. All authors read and approved the final manuscript.
